# Two-Stage Cross-Hand Transfer: A Case Report

**DOI:** 10.7759/cureus.34001

**Published:** 2023-01-20

**Authors:** Tahseen Cheema, Husnain Khan, Nur Ul Ain

**Affiliations:** 1 Orthopedic Surgery, National Orthopedic and General Hospital, Bahawalpur, PAK; 2 Plastic and Reconstructive Surgery, Holy Family Hospital, Rawalpindi, PAK

**Keywords:** cross-hand transfer, reconstructive surgery, plastic surgery, hand rehabilitation, finger transfer, thumb transfer, hand surgery, hand reconstruction, poliomyelitis, amputation

## Abstract

This is a case report of a patient who presented with amputation of all digits of the left hand just distal to the metacarpophalangeal joint level due to a fodder cutter injury one year ago. There was poliomyelitis of the right hand since childhood. The patient was managed at the National Orthopedic Hospital, Bahawalpur, in 2014-2015. The surgery was planned in two stages. In stage 1, only the thumb transfer from the opposite hand was done. Stage 2 was performed three months later, in which the transfer of three digits was done from the opposite hand. Follow-up was done at one month, four months, and one year after surgery. The patient had a good recovery and is able to perform daily activities of life with excellent cosmetic results.

## Introduction

Non-functioning of both hands either due to traumatic amputations, congenital deformities, or infective processes results in severe disability of human life. Restoration of hand functions by transfer of an intact finger from a normal hand to a damaged hand was first proposed by Morrison et al. in 1982 [1]. They transferred the ring finger from the normal hand to the injured hand having amputations of all digits (metacarpal hand). Other authors have reported transfer from the opposite hand primarily in the case of an absent thumb, utilizing a damaged digit from the contralateral side or two digits [2,3]. Cross-hand transfer in the case of bilateral upper extremity amputations [4], traumatic injury of one hand and contralateral cerebrovascular spasticity in the other hand have been reported [5]. Although there was some improvement in hand function in all these reports, the hand function was still not optimal as the thumb was on the ulnar border of the hand. Moreover, the obvious problems were cosmetic concerns due to a lack of the thumb in its proper position and complicated techniques to place the thumb in its original position.

## Case presentation

**Figure 1:**
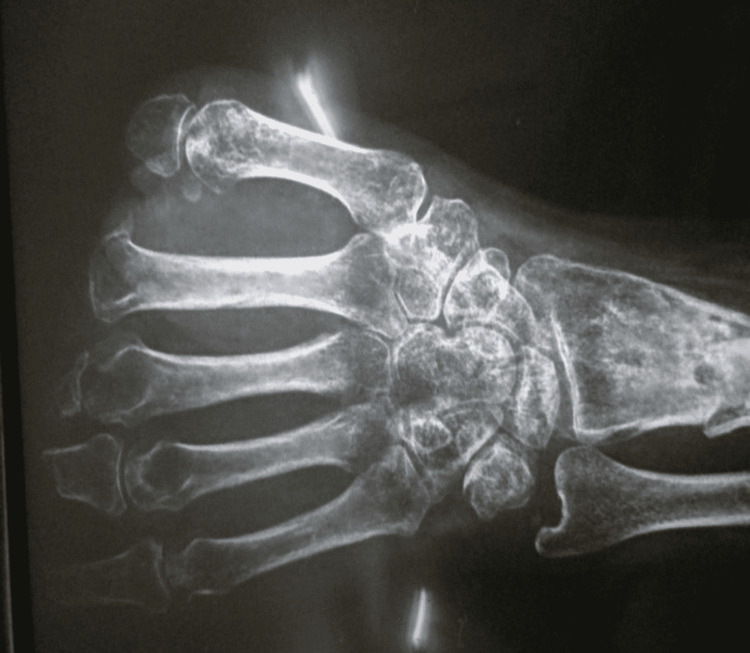
Radiograph of the left hand depicting the level of amputations.

**Figure 2:**
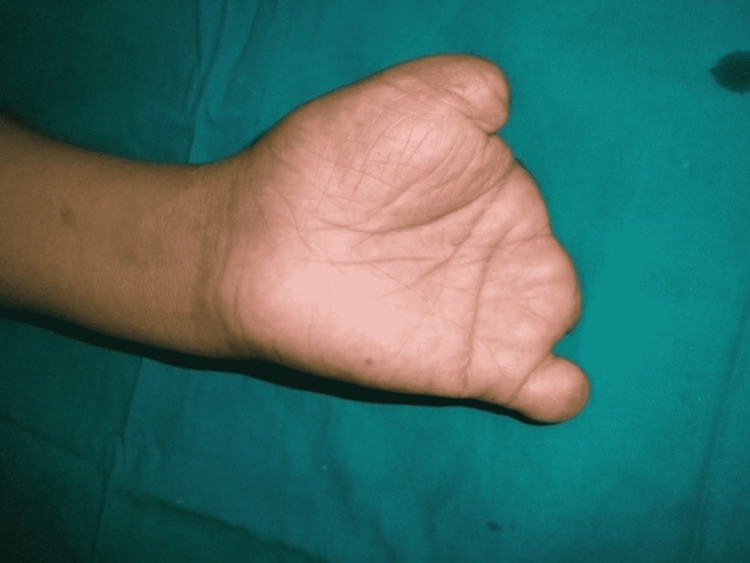
Level of amputations of the left hand.

A 35-year-old male, a left-handed farmer by occupation, presented in 2014 at the senior author’s institution. There was an amputation of all digits of the left hand due to a fodder cutter injury 11 months ago. The amputation at the thumb, middle, ring, and little finger was just distal to the metacarpophalangeal joint with preservation of some part of the base of the proximal phalanx. The index finger was amputated at the level of the metacarpophalangeal joint (Figures 1, 2).

**Figure 3:**
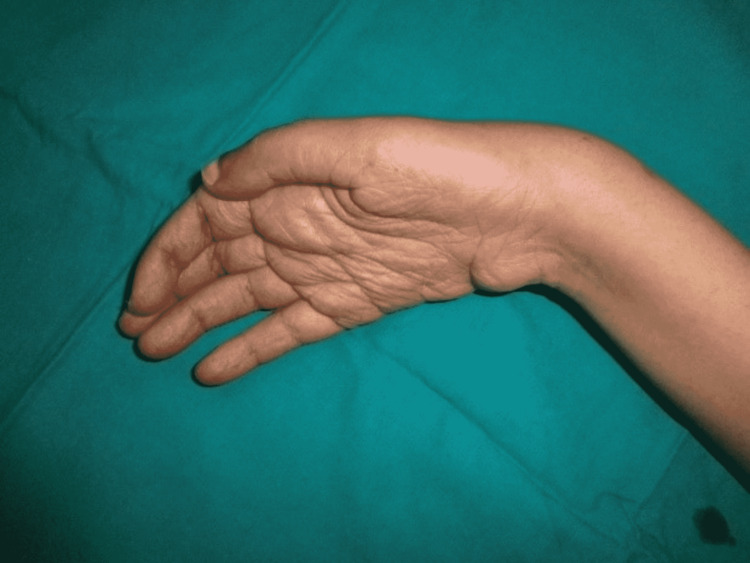
Poliomyelitis of the right hand.

After primary closure at some other hospital, the stump of the hand and thumb was healed. There was an incidental finding of poliomyelitis of the right hand with a good passive range of motion. This bilateral deformity resulted in the severe disability of the patient (Figure 3).

**Figure 4:**
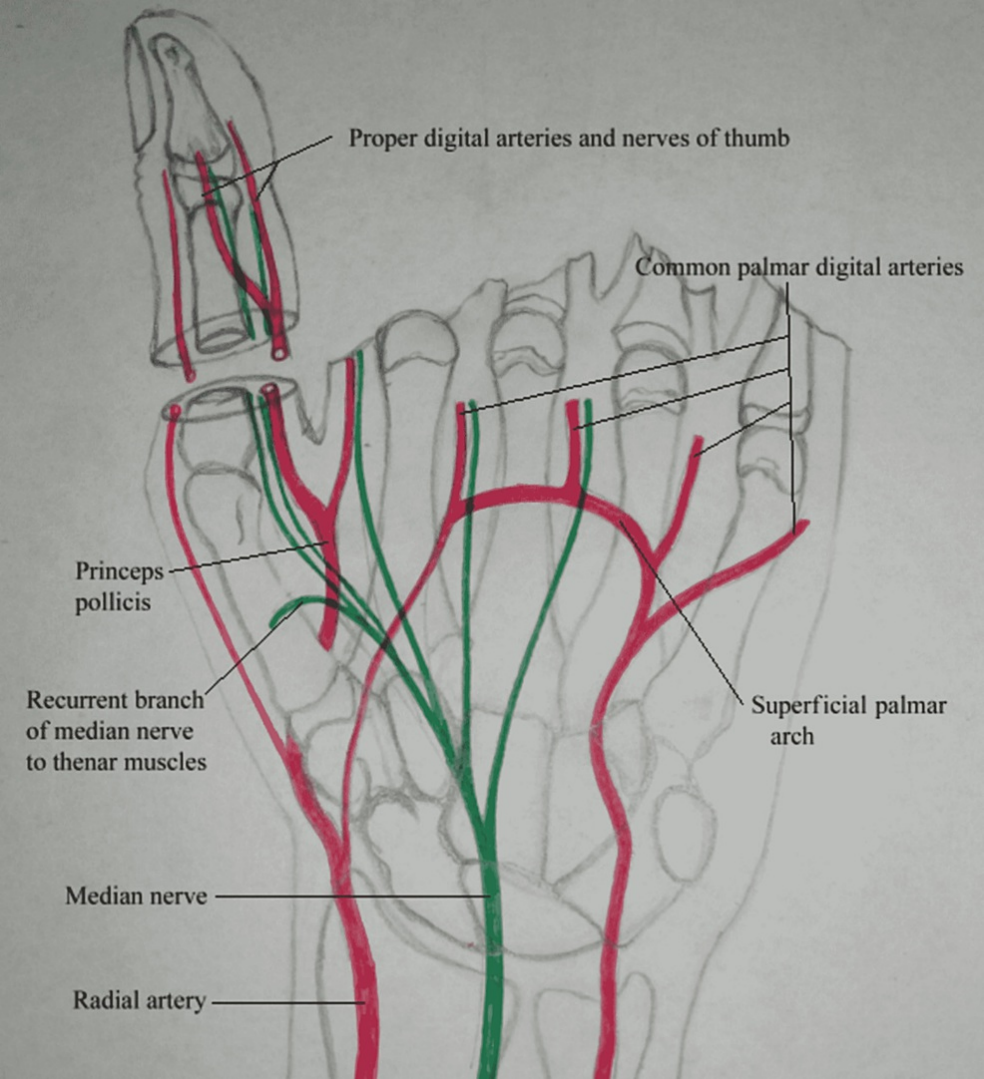
Schematic representation of stage 1.

**Figure 5:**
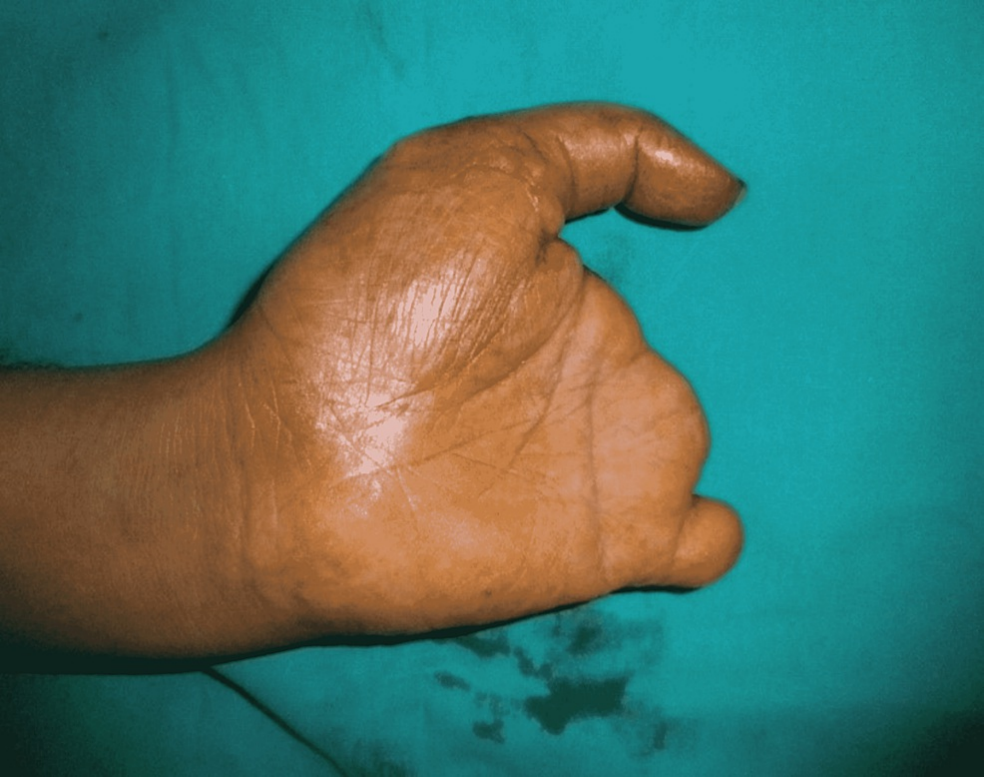
Replanted thumb.

We planned to operate this patient in two stages. In stage 1, we transferred the thumb from the paralytic hand to the amputated hand. Radial artery, cephalic vein, and digital branches of the median nerve to the thumb were used as donor neuro-vascular components. Skeletal fixation was done distal to the metacarpophalangeal joint, preserving the joint. After adjusting the tension, the flexor pollicis and extensor pollicis longus were repaired (Figures 4, 5).

**Figure 6:**
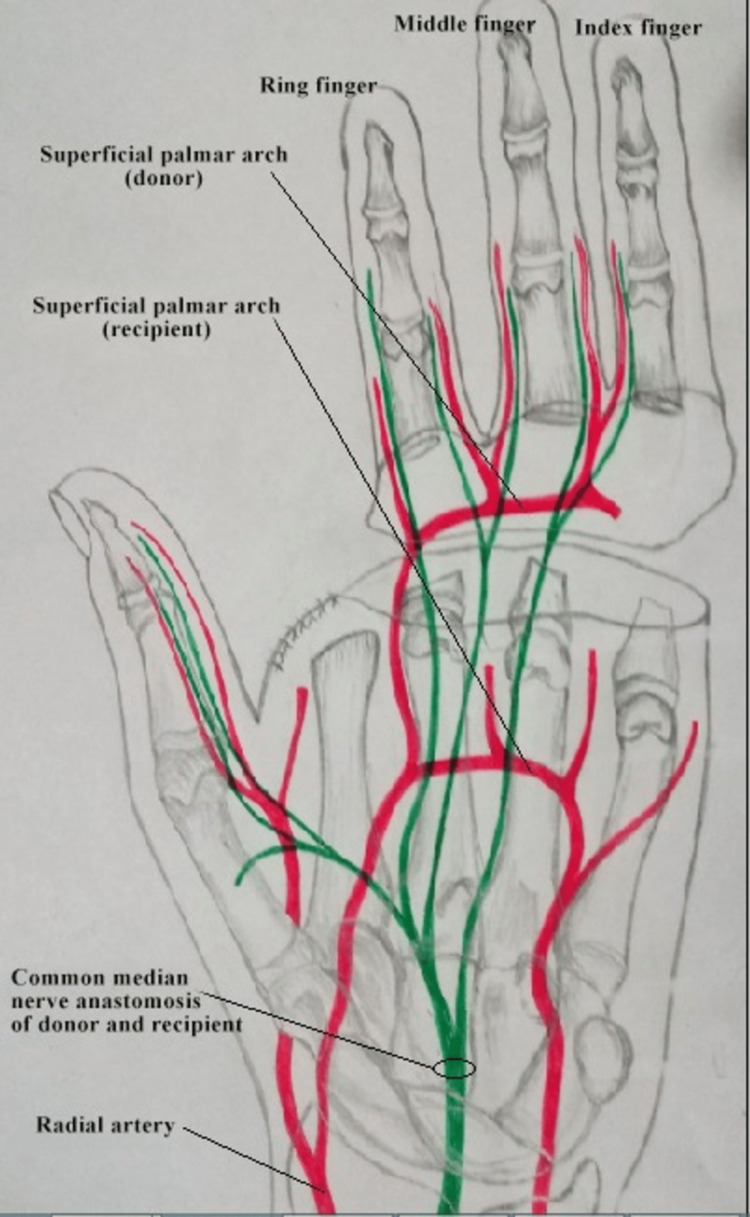
Schematic representation of stage 2.

**Figure 7:**
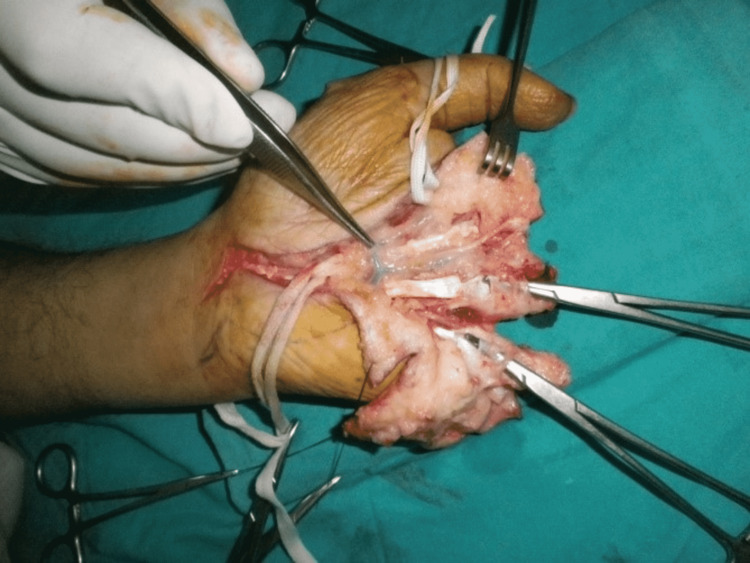
Exploration on a volar surface.

**Figure 8:**
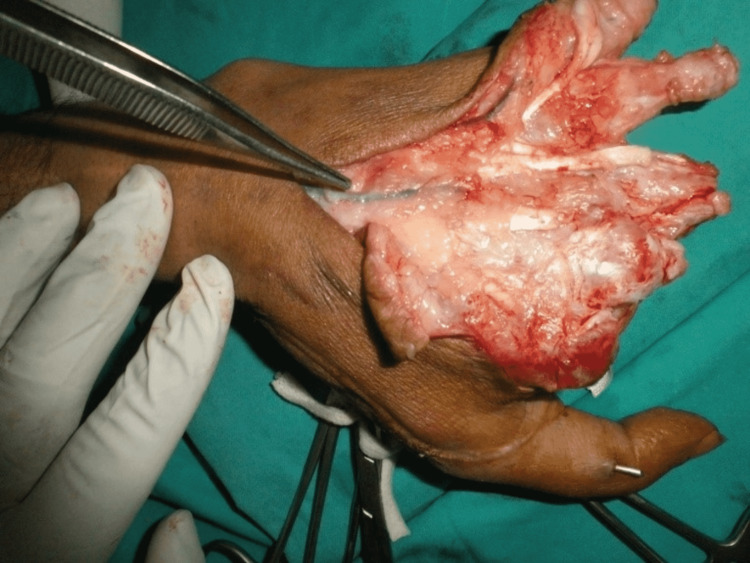
Exploration on a dorsal surface.

**Figure 9:**
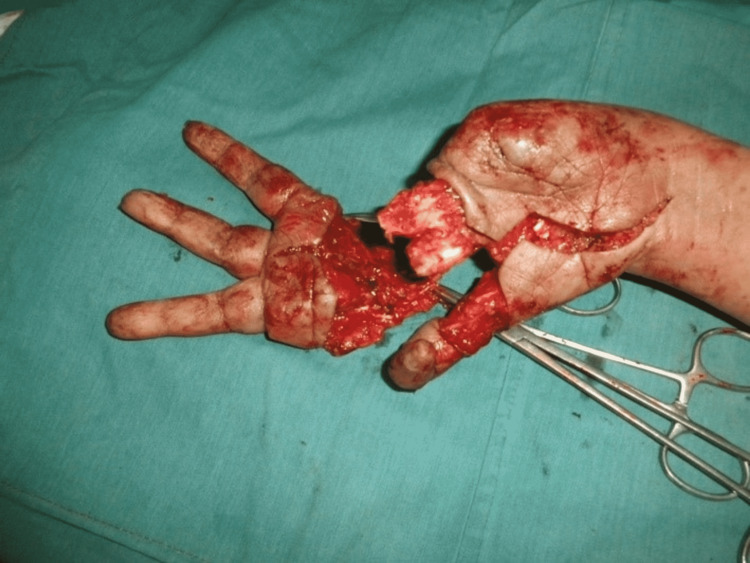
Three-finger transfer.

**Figure 10:**
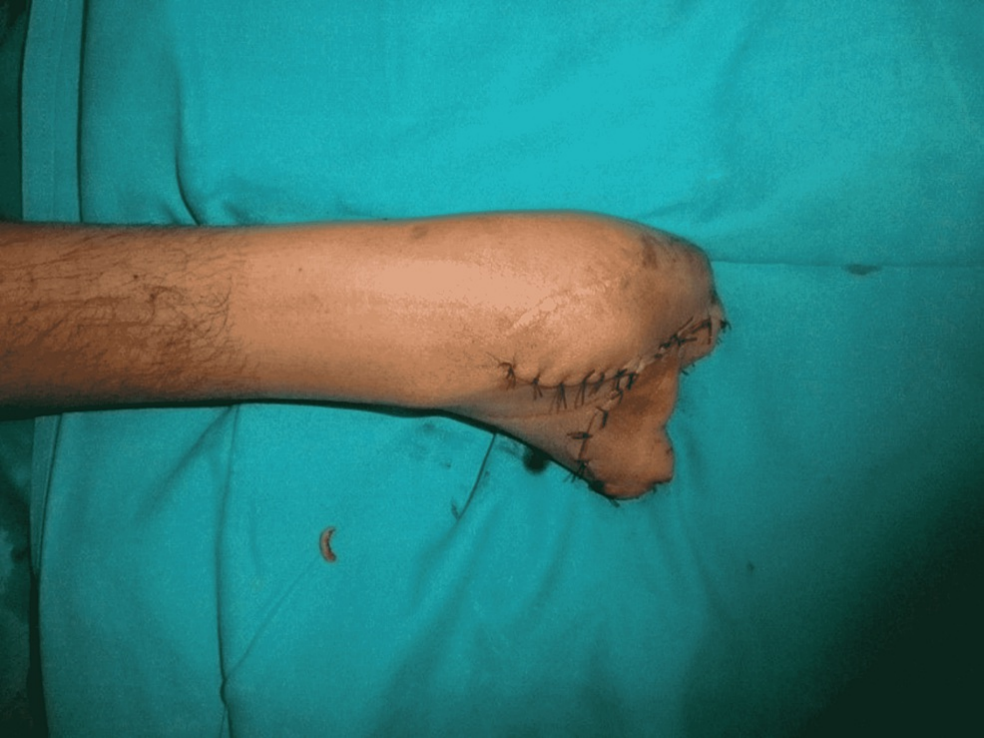
Donor site closure.

Three months later, when the thumb had attained normal function and position, we performed stage 2 (Figures 6-10). Our plan was to replace the ring finger of the paralytic hand to the middle finger, the middle finger to the ring finger, and the index finger at the place of the little finger of the traumatic hand. The little finger skin fillet was used to cover the stump of the paralytic hand. The rest of the structure alignment was done as follows: (1) Skeletal fixation: osteotomies were done in the paralytic hand according to the amputation of the amputated hand. Kirchner wires were used for fixation. These were all distal to metacarpophalangeal joints, keeping in view that there will be no discrepancy in length at the recipient site. (2) Vascular anastomosis: superficial palmar arch of the paralytic hand was taken and anastomosed with the second common digital artery. The dorsal carpal venous arch was anastomosed with the common dorsal venous arch. (3) Nerve anastomosis: neurorraphy of both donor and recipient common sensory fascicle of the median nerve (just distal to the origin of the recurrent motor branch) was performed. (4) Extrinsic tendon reconstruction: both flexors and extensors of three digits (index, middle, and ring) were repaired according to the modified Kessler repair technique.

**Figure 11:**
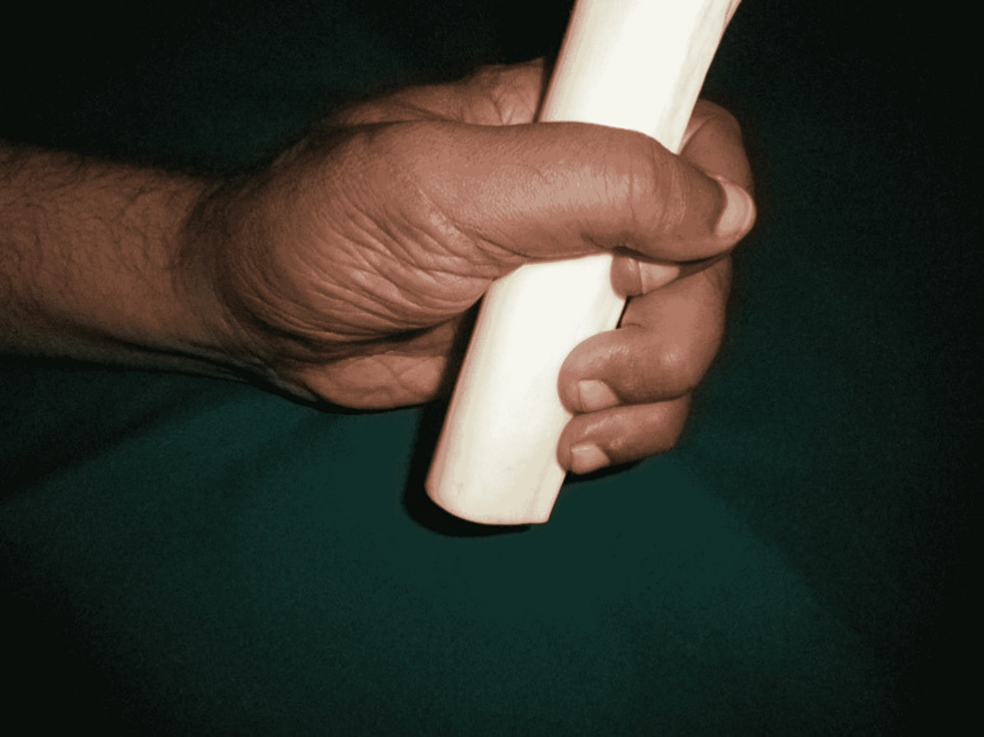
Hand movements after one year. Grasping an object.

**Figure 12:**
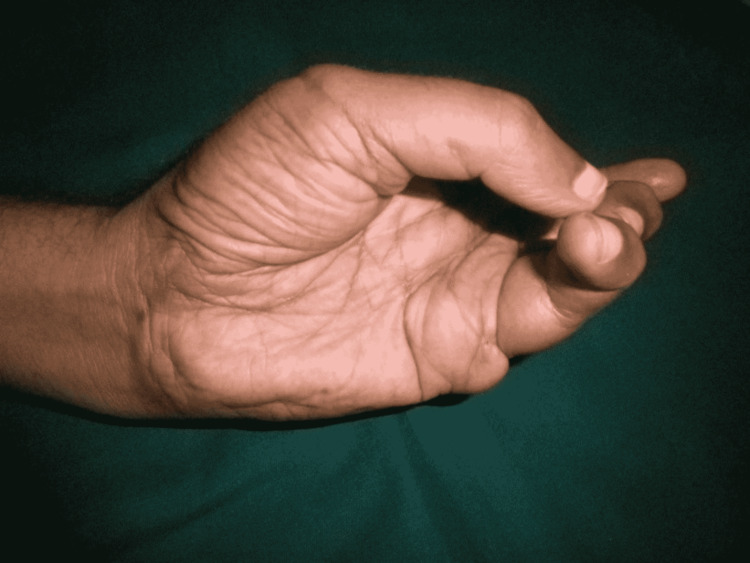
Hand movements after one year. Opposition movement.

**Figure 13:**
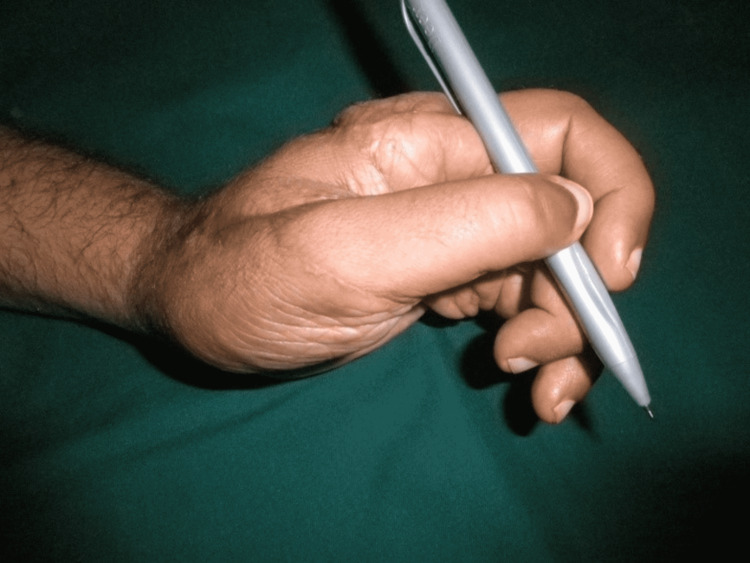
Hand movements after one year. Holding a pen.

The patient remained admitted for one week postoperatively with monitoring of the replanted part. The follow-up was done at one month, four months, and one year after surgery. The patient showed 5/5 muscle power in the replanted digit and performed daily activities of life. The static two-point discrimination at all digits was 5 mm after one year (Figures 11-13).

## Discussion

Bilateral upper limb injuries resulting in complete disability are difficult conditions to treat and have tested surgeons over the years. These injuries may result after acute trauma or congenital and infected diseases. In the case of amputation, the use of the amputated part of one limb to other limb has been described [5]. Moreover, there are certain instances of using the paralytic limb for transfer to the other side with amputation or injury. The transfer of the upper extremity has been reported in cases of amputation of an extremity and brachial plexus injury or palsy due to cerebrovascular accidents of the other extremity [6]. In the case of a brachial plexus injury on the left side, a free microvascular cross-hand transfer of the right hand was performed to the left distal carpus [7]. Cross-transfer of the right hand to the left distal forearm stump has been reported in a long-standing left radial-carpal amputee who had developed a contralateral cerebrovascular accident with severe right-sided spasticity. In this case, the thumb column of the right hand was transposed to the ulnar side of the hand, which had its shortcomings [8]. Reagan described a rare case of cross-arm transfer [9]. Cross-transfer has been described for the foot as well, where elective cross-transfer of the left foot was done to the right distal tibial stump after a right Syme amputation [10]. These unfortunate situations are very rare, and few case reports of cross-hand transplantation have been documented. In all these reported cases with transfer at the level or proximal to wrist, the radius and ulna were fixed to the ulna and radius, respectively, resulting in complete blocking of supination and pronation. In cases where complete hand transfer was done distal to the wrist, the thumb was situated on the ulnar border. All these previous cases resulted in severe functional and cosmetic limitations. In our technique, we took care of cosmetic as well as functional problems. We found out that in the case of the single-stage transfer, we would face similar problems as mentioned above; therefore, we performed our surgery in two stages. This planning resulted in a thumb in its natural position, i.e., on the radial aspect of the hand. The use of the index, middle, and ring finger of a paralytic hand to little, ring, and middle finger, respectively, has resulted in a naturally appearing hand with a wide first web space. Moreover, strengthening the ulnar border of the hand has helped the patient who was a farmer by occupation. Our technique has some similarities with Pedro and colleagues' technique, in which they also perform the cross-hand transfer in two stages. However, their technique was a bit complicated, and the osteotomies were done proximally to the wrist with fixation of the radius and ulna by plates [8].

## Conclusions

The advantages of our technique were single arterial, venous, and sensory anastomoses. This resulted in the preservation of operative time. Furthermore, we performed our technique in stages, which resulted in a more natural position of the thumb. This provided both functional and esthetic benefits. Moreover, one of the drawbacks we observed in our technique was that the ulnar border of the ring finger at the donor's hand (supplied by the ulnar nerve) has now become the radial border of the index finger at the recipient site, which initially lacks sensation. However, after one year, the patient developed a protective sensation along the radial border, possibly due to sensory overlap. We concluded that our technique was a comparatively simple, time-efficient, yet effective solution for the issue at hand.
